# RNA-Seq Data-Mining Allows the Discovery of Two Long Non-Coding RNA Biomarkers of Viral Infection in Humans

**DOI:** 10.3390/ijms21082748

**Published:** 2020-04-15

**Authors:** Ruth Barral-Arca, Alberto Gómez-Carballa, Miriam Cebey-López, María José Currás-Tuala, Sara Pischedda, Sandra Viz-Lasheras, Xabier Bello, Federico Martinón-Torres, Antonio Salas

**Affiliations:** 1 Unidade de Xenética, Instituto de Ciencias Forenses (INCIFOR), Facultade de Medicina, Universidade de Santiago de Compostela, 15782 Galicia, Spain; barralarcaruth@gmail.com (R.B.-A.); Alberto.Gomez.Carballa@sergas.es (A.G.-C.); Miriam.Cebey.Lopez@sergas.es (M.C.-L.); mjcurras@gmail.com (M.J.C.-T.); sara.pischedda01@gmail.com (S.P.); sandravizlasheras@gmail.com (S.V.-L.); xbello@gmail.com (X.B.); 2 GenPoB Research Group, Instituto de Investigaciones Sanitarias (IDIS), Hospital Clínico Universitario de Santiago (SERGAS), 15706 Galicia, Spain; 3 Genetics, Vaccines and Pediatric Infectious Diseases Research Group (GENVIP), Instituto de Investigación Sanitaria de Santiago (IDIS) and Universidad de Santiago de Compostela (USC), 15706 Galicia, Spain; Federico.Martinon.Torres@sergas.es; 4 Translational Pediatrics and Infectious Diseases, Department of Pediatrics, Hospital Clínico Universitario de Santiago de Compostela (SERGAS), 15706 Galicia, Spain

**Keywords:** biomarkers, RNA-seq, lncRNA, virus, machine learning

## Abstract

There is a growing interest in unraveling gene expression mechanisms leading to viral host invasion and infection progression. Current findings reveal that long non-coding RNAs (lncRNAs) are implicated in the regulation of the immune system by influencing gene expression through a wide range of mechanisms. By mining whole-transcriptome shotgun sequencing (RNA-seq) data using machine learning approaches, we detected two lncRNAs (ENSG00000254680 and ENSG00000273149) that are downregulated in a wide range of viral infections and different cell types, including blood monocluclear cells, umbilical vein endothelial cells, and dermal fibroblasts. The efficiency of these two lncRNAs was positively validated in different viral phenotypic scenarios. These two lncRNAs showed a strong downregulation in virus-infected patients when compared to healthy control transcriptomes, indicating that these biomarkers are promising targets for infection diagnosis. To the best of our knowledge, this is the very first study using host lncRNAs biomarkers for the diagnosis of human viral infections.

## 1. Introduction

The majority of erroneous antibiotic prescriptions occur in virus-infected patients, for which antibiotics offer no benefit except for mixed viral/bacterial infections [1,2]. Inadequate use of antibiotics not only is an economic burden for the sanitary systems—especially for developing countries—but also it increases the risk of adverse events and, more importantly, the emergence of resistant bacteria [2]. According to the World Health Organization (WHO), antibiotics overuse is one of the biggest threats to human health nowadays [3]. The development of a fast and accurate diagnostic testing to early distinguish viral from bacterial infections in clinical settings and hospitals would facilitate a reduction in the overuse of wide-spectrum antibiotics, helping physicians make the right decisions and fight the appearance of antibiotic-resistant bacteria.

The gold standard procedure to detect the presence of bacterial infections is bacterial culture, but this technique has important limitations. Firstly, obtaining results from cultures usually takes 48–72 h, a timeframe that might be inadequate for decision-making in terms of antibiotic prescriptions to children with suspected infection. Secondly, bacterial cultures have limited sensitivity, to the extent that, e.g., failure to detect causal microorganisms occurs in 50% of pneumonia patients in critical care units [4,5]. According to Rozon et al. [6], the sensitivity and specificity of a Gram stain from a good-quality sample in the diagnosis of pneumococcal pneumonia and *H. influenzae* ranged 57–82%. Besides, samples obtained from accessible sites (e.g., blood) can be useless for pathogen detection in cases where the infection is located in inaccessible anatomical sites. Third, viruses are usually detected in healthy children, and also in children with bacterial infections. For instance, in a large international collaborative study, Martinón-Torres et al. [7] recently reported that pathogen detection has been of limited help to distinguish viral from bacterial infection.

In 2007, Ramilo et al. [8] showed that RNA from blood leukocytes of children with life-threatening viral or bacterial infections bears host-pathogen specific transcriptional signatures. This finding represented a shift of paradigm in the study of infectious diseases since, for the first time, the host, and not the pathogen, became the focus of attention. Since then, RNA analysis has arisen as a powerful screening tool to find diagnostic biomarkers that may be used to develop new tests that overcome the limitations of bacterial culture. Recently, several studies have been exploring host-specific transcriptomic biomarkers that may allow distinguishing between viral and bacterial infections or pathogen-specific signatures [9,10,11,12,13,14,15,16]. Related to transcriptional signatures, there are also several studies relating host genetic susceptibility factors to infectious diseases [17,18,19,20,21].

Thanks to the recent advances of transcriptomics and bioinformatics, new long non-coding RNAs (lncRNAs) are being discovered and characterized each year. LncRNAs are transcripts with lengths exceeding 200 nucleotides that do not translate into proteins. Despite their abundance, the role of these molecules in infectious diseases has not been studied yet.

The present study sought viral lncRNAs host biomarkers that allow discriminating viral from healthy controls and assessed whether these genes could be the basis for a new diagnostic tool that could be implemented in the clinical routine.

## 2. Results

### 2.1. Evaluation of lncRNAs in the Context of Viral Infection

Our machine learning model detected two lncRNAs of viral infection, namely, ENSG00000273149 and ENSG00000254680. According to Ensembl (www.ensembl.org), ENSG00000273149 is a recently described transcript located in chromosome 13 and anti-sense transcribed with respect to the translationally-controlled tumor protein gene (*TPT1*). As ENSG00000273149 is a recently described transcript not much is known about it, except for the fact that is has been described to be associated with vascular stenosis [22]. ENSG00000254680 is located in chromosome 11; since it is also a recently described transcript, there is no published information about it.

**Figure 1:**
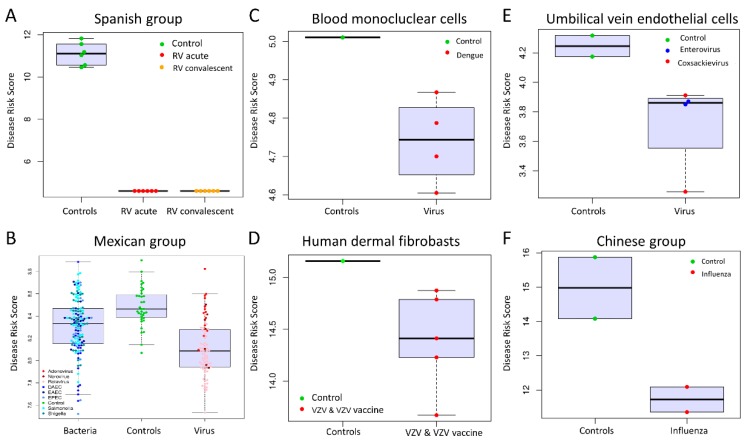
Classification performance based on the two lncRNA VS, considering different viral pathogens and studies. Box and whisker plots of VS values: (**A**) Discovery group (Spanish group); (**B**) Test group GSE69529; (**C**) External validation group GSE98859; (**D**) External validation group GSE121385; (**E**) External validation group GSE94551; and (**F**) External validation group PRJNA230906. For all box plots, the horizontal lines in boxes indicate median of the groups; the lower and upper sides of boxes interquartile ranges and the whiskers < 1 times the interquartile range. On the *x*-axis is the sample status and on the *y*-axis the VS calculated as log_2_ of the sum of counts of our 2-transcript diagnosis model. The acronyms stand for: RV (Rotavirus), VZV (Varicella Zoster Virus), diffuse-adhering *Escherichia coli* (DAEC), enteropathogenic *Escherichia coli* (EPEC), and enteroaggregative *Escherichia coli* (EAEC).

We evaluated if patients clustered according to their disease status (viral infection, bacterial infection, and healthy controls) when applying the Viral Score (VS; see methods) to different groups represented by several pathogens and tissues. For this comparison, we generated boxplots with overlapping one-dimensional scatter plots, where high VS values indicate healthy status, whereas a lower VS indicates viral infection. This analysis showed that in most of the scenarios (Figure 1), there are significant differences in the VS of affected children by viral infection versus healthy controls (1A,1C,1D). The only exception occurs in the Mexican group (Figure 1B) where the boxplot shows some overlap in the first and third quartiles (75th percentile) of the boxplots for virus-infected patients versus controls. This could be explained by the fact that the Mexican samples represent a mixed group of children with mild and severe infections [23].

**Figure 2:**
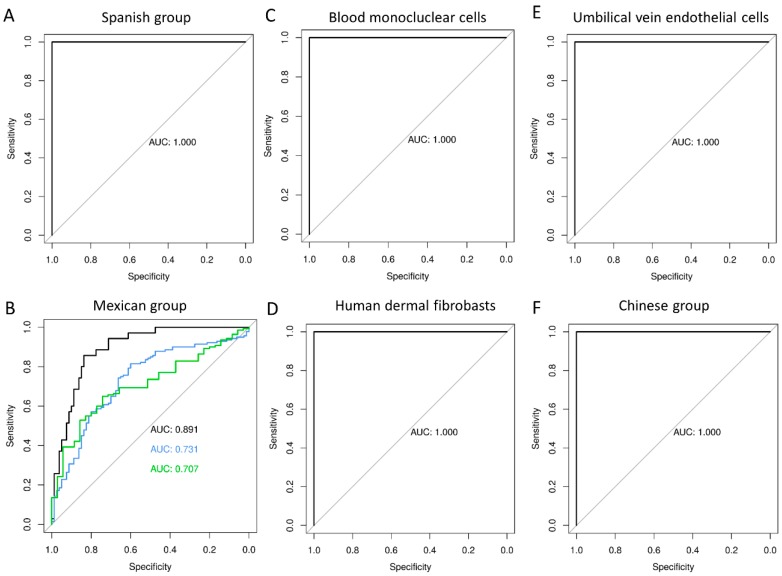
Evaluation of VS test performance. AUC (Area Under the Curve) values are provided in Table 1. ROC curves for VS: (**A**) Discovery group (Spanish group); (**B**) Test group GSE69529; (**C**) External validation group GSE98859; (**D**) External validation group GSE121385; (**E**) External validation group GSE94551; and (**F**) External validation group PRJNA230906. For all figures, the horizontal lines in boxes indicate median of the groups; the lower and upper sides of boxes interquartile ranges and the whiskers < 1 times the interquartile range. On the *x*-axis is the sample status and on the *y*-axis the VS calculated as log_2_ of the sum of counts of our 2-transcript diagnosis model.

The diagnostic accuracy of the 2-lncRNA test was evaluated by ROC analysis (Figure 2, Table 1). For all the tested scenarios, the ROC curves indicate that the accuracy of the test based on the two lncRNAs is very high (AUC > 89%) when comparing viral infection against healthy controls in all the datasets. The accuracy drops moderately when comparing bacterial versus viral infection (AUC = 73%), and it drops slightly more when comparing bacteria versus controls (AUC = 70%):

1. Influenza versus controls (PRJNA230906), AUC = 100% (CI95%: 100–100%)
2. Dengue versus controls (GSE98859), AUC = 100% (CI95%: 100–100%)
3. Enterovirus/coxsakie virus (GSE94551) versus controls, AUC = 100% (CI95%: 100–100%)
4. RV versus controls (PRJNA325575), AUC = 100% (CI95%: 100–100%)
5. RV/norovirus/adenovirus versus controls (GSE69529), AUC = 89% (CI95%: 83–95%)
6. *E. coli/Salmonella/Shigella* versus controls (GSE69529), AUC = 70% (CI95%: 62–79%)
7. *E. coli/Salmonella/Shigella* versus RV/norovirus/adenovirus, AUC = 73% (CI95%: 66–80%)
8. Varicella zoster versus controls (GSE121385), AUC = 100% (CI95%: 100–100%).

### 2.2. The efficiency of lncRNAs *versus* Other Minimal Transcriptomic Signatures

We compared the performance of the two lncRNAs signature with the 2-transcript signature (*IFI44L* and *FAM89*) described by Herberg et al. [13] using microarray data, which was also identified as a minimum transcriptomic signature for distinguishing between viral and bacterial infections.

We found that the power to differentiate between viral and bacterial infection in our groups was very similar for the two signatures: AUC_lncRNA_ = 0.7311 [CI95%: 0.6615–0.8006] versus AUC_*IFI44L* + *FAM89*_ = 0.745 [CI95%: 0.6743–0.8157]. However, when comparing against healthy controls the performance of the two lncRNA is slightly better (Table 1).

We also evaluated the possibility of combining the information provided by the two lncRNAs with the expression level of *IFI44L* alone [24], and *IFI44L* + *FAM89A*. We found that the prediction accuracy in terms of AUC did not improve significantly (Table 1):

1. Influenza versus controls (PRJNA230906), AUC_lncRNAs + *IFI44L* + *FAM89A*_ = 100% (CI95%: 100–100%),
2. Dengue versus controls (GSE98859), AUC_lncRNAs + *IFI44L* + *FAM89A*_ = 100% (CI95%: 100–100%),
3. Enterovirus/coxsakie virus (GSE94551) versus controls, AUC_lncRNAs + *IFI44L* + *FAM89A*_ = NE; meaning that at least one of the genes of the signature was not expressed (NE) and the score could not be calculated,
4. RV versus healthy controls (PRJNA325575), AUC_lncRNAs + *IFI44L* + *FAM89A*_ = 100% (CI95%: 100–100%),
5. RV/norovirus/adenovirus versus controls (GSE69529), AUC_lncRNAs + *IFI44L* + *FAM89A*_ = 85% (CI95%: 77–92%),
6. *E. coli*/Salmonella/Shigella versus controls (GSE69529), AUC_lncRNAs + *IFI44L* + *FAM89A*_ = 75% (CI95%: 68–82%),
7. *E. coli*/Salmonella/Shigella versus RV/norovirus/adenovirus, AUC_lncRNAs + *IFI44L* + *FAM89A*_ = 68% (CI95%: 58–78%),
8. *Varicella zoster* versus controls (GSE121385), AUC_lncRNAs + *IFI44L* + *FAM89A*_ = 100% (CI95%: 100–100%).

In agreement with recent findings [24], the addition of *FAM89A* to the signature did not significantly improve (and even worsened) the signature performance.

## 3. Discussion

According to the Encyclopedia of DNA Elements (ENCODE; [25]) pilot project, most of the human genome (74–93%) transcribes in multiple RNAs, and the vast majority of them does not display protein coding capacity [26,27]. New sequence technologies (e.g., RNA-seq) have recently led to the discovery of many previously unknown no protein-coding lncRNAs. Even though these RNAs are encoded by an important proportion of the genome, most of their functions are still unknown [28]. This is because, unlike mRNAs or proteins, presently the function of lncRNAs cannot be predicted by looking at the sequence or structure. Their function is most likely related to chromatin modification, transcriptional regulation, and post-transcriptional regulation [29]. It is currently widely accepted that lncRNAs are involved in biological and pathological processes such as X dosage compensation in mammals, regulation of the immune response, gene imprinting, regulation of the cell cycle, telomere length, etc. [28,30]. The strongest evidence to support the role of host lncRNAs in the regulation of innate and adaptative immune system has come from animal models of infection and/or disease [30]. Thus, Peng et al. [31] observed that coronavirus infection in lung tissue altered globally the lncRNAs levels in several mouse strains. A few years later, experiments by Gomez et al. [32] carried out in transgenic mice infected with *Salmonella* and Theiler’s virus found that the upregulation of the lncRNA *NeST* increased the clearance of *Salmonella* infection, but also reduced resistance to the mouse Theiler’s picornavirus. According to their results, *NeST* induces interferon γ (*IFN*-γ), a cytokine that is critical for innate and adaptive immunity against viral, some bacterial and protozoal infections expression in T cells. Almost simultaneously, Zhang et al. [33] reported that the knockdown of the lncRNA *NEAT1* enhances HIV-1 virus production. A year later, Imamura et al. [34] found that the *NEAT1* promoted *IL8* expression in response to viral infection in human cells. Nevertheless, it has been reported that a major problem in the study of lncRNA in animal models is the lack of evolutionary conservation of the lncRNAs between species, which constitutes a major barrier in extrapolating results from animal models to humans [30].

There is a growing body of evidence suggesting that lncRNAs play a role in the host susceptibility and defense against viral infections [30]. The present study used a data-driven approach based on comparing the whole transcriptome of healthy children and children suffering a viral infection. Following this method, we found two lncRNAs (ENSG00000254680 and ENSG00000273149) downregulated upon viral infection whose expression level can be used to detect the presence of viral infection. These two lncRNAs perfectly distinguish healthy controls from viral infected patients in a broad sense, which suggests that they may play a role in the host defense against viral infections or in the host susceptibility to infection. These two lncRNA molecules are abundant enough to be detected in a variety of different biological scenarios as seen in boxplots of Figure 2.

Little information is available for these two lncRNAs. The antisense transcript ENSG00000273149 is also known as AL138963.3, and it is located in chromosome 13 (http://www.ensembl.org/Homo_sapiens/Gene/Summary?g=ENSG00000273149;r=13:45340039-45341183;t=ENST00000610057). To the best of our knowledge, the only reference about it in the literature describes its upregulation in the tissue of arteriovenous fistula [22].

The lncRNA ENSG00000254680 (also known as AC079329.1) was found to be downregulated in viral infection. It is located in chromosome 11, and it also has been recently described (http://www.ensembl.org/Homo_sapiens/Gene/Summary?g=ENSG00000254680; *r* = 11:12261426-12263173; *t* = ENST00000527288). The only reference existing in the literature for this lncRNA corresponds to a patent describing methods to reduce T cells exhaustion, indicating that AC079329.1 is downregulated by c-Jun gene expression [35]. This description is in line with our observation because the c-Jun protein has proven to be very similar to an avian sarcoma virus 17 protein, which directly recognizes specific DNA sequences to regulate its expression [36].

The possibility of using only a few transcripts to diagnose viral infections renders these two lncRNAs attractive biomarkers to design a rapid point of care test, or even a qPCR-based assay, applicable in hospital settings [24,37]. It is worth noting that there already exists a commercial test that uses lncRNAs for diagnosis and prognosis purposes: the ExoDx™ Prostate (IntelliScore) urine test detects three biomarkers associated with aggressive prostate cancer, one of them is a lncRNA transcript [38,39].

Even so, the use of expression biomarkers, such as lncRNAs in diagnosis settings, is still limited because the RT-qPCR, the gold standard method for gene expression measurement, requires access to a laboratory, a thermocycler, and relatively sophisticated data analysis [24,38]. Nevertheless, recent advances in transcriptomic and portable technologies (e.g., Nanopore [4]), suggest that in the next few years we will probably see an explosion in the use of mRNAs and lncRNAs expression signatures as a point of care diagnostic tools for many pathologies [40,41,42,43].

In all of the phenotypic scenarios examined, our results suggest that the two described lncRNAs provide a signal strong enough to identify viral infections in a broad sense. The major limitation of the present study is the low sample size used to build the classification model; unfortunately, there are only a few RNA-seq studies on infectious diseases available [23,26]. Despite the limited amount of information available, our sample sizes are in line with other RNA-seq studies [44]. It is important to note that our meta-analysis could not benefit from the vast datasets publicly available on commercial microarrays because the lncRNAs found in our study were not included in these datasets.

Further research is thus needed to evaluate the accuracy of these non-coding biomarkers in different clinical scenarios, including different severities, the evolution of the signal according to the time from the onset of the disease, more microorganisms, scenarios of co-infection [45], etc. However, taken together, the present results suggest that the 2-lncRNAs signature has a good prediction capacity, comparable to the coding RNAs described in the literature to date.

Finally, the fact that the lncRNAs signature shows very good performance in different human population groups, tissues, and families of virus (such as *Herpesviridae, Flaviviridae*, *Orthomyxoviridae*, and *Reoviridae*), suggests that these biomarkers are most likely related to a molecular mechanism related to host response or susceptibility against viral infection that is well-preserved from an evolutionary point of view. Unravelling their role may allow the discovery of unknown pathogenic pathways and drug targets, which might eventually lead to the discovery of wide-spectrum antiviral drugs.

## 4. Conclusions

The present study represents a stepping stone to the ultimate goal of understanding virus–host interaction mechanisms in viral parthenogenesis. As far as we are aware, this is the first study to have found host lncRNAs with potential as viral diagnostic biomarkers or therapeutic targets. We have shown that two lncRNAs are downregulated during viral infections in blood and different types of cells compared to healthy control samples. Moving the present results to translational medicine and the future use of these markers as diagnostic tools in clinical settings would require preliminary testing on a wider range of well-selected samples from cultured cells and clinical samples. Moreover, further functional studies are needed to unravel the mechanisms by which these lncRNAs act during the infection.

## 5. Material and Methods

### 5.1. Sample Groups

**Figure 3:**
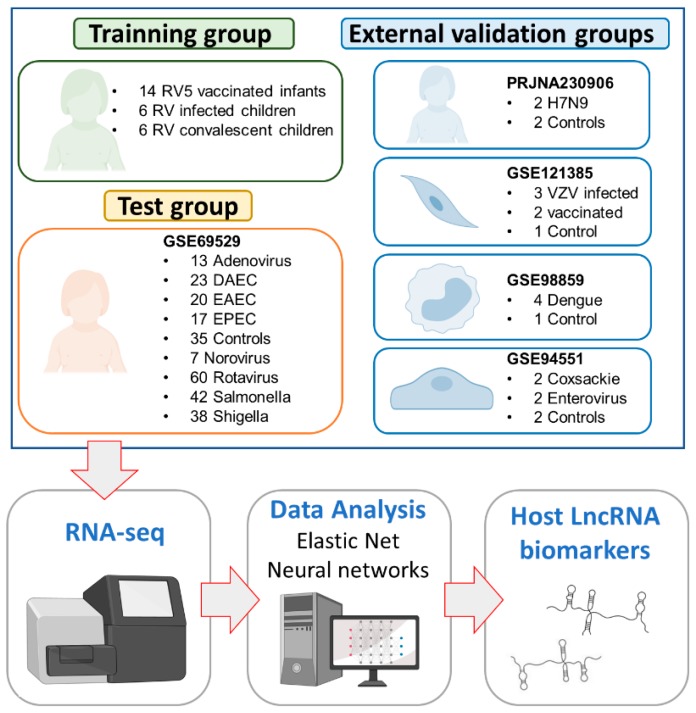
Diagram of the study design. The acronyms stand for: Rotateq^®^ (RV5); rotavirus (RV); diffuse-adhering *Escherichia coli* (DAEC), enteropathogenic *Escherichia coli* (EPEC) and enteroaggregative *Escherichia coli* (EAEC), and Varicella Zoster Virus (VZV).

Six groups of patients or infected cells and uninfected controls were analyzed in the present study (Figure 3).

The Spanish group consists of 18 western European children [46]. Blood samples were collected between 2013–2014 at the Hospital Clínico Universitario of Santiago de Compostela (Galicia; northwest Spain); they include: (i) 6 rotavirus (RV)-infected children that required medical attention at two different time-points, namely, acute (during medical attendance) and convalescent phases; and (ii) 6 healthy controls (with all the vaccines of the Spanish immunization schedule up to date but no RV vaccine); Table S1. A subset of these controls and infected children was previously explored in a separate study [26].

The Mexican group consists of 255 blood samples of healthy controls (*n* = 35), and patients with acute diarrhea caused by different bacterial/viral pathogens: RV (*n* = 60), norovirus (*n* = 7), adenovirus (*n* = 13), *Salmonella* (*n* = 42), *Shigella* (*n* = 38) and different strains of *E. coli* (*n* = 60); Gene Expression Omnibus (GEO) accession number: GSE69529 (more detailed information about the samples is provided in [47]).

The Chinese group comprises four blood samples collected from patients with H7N9 infection (*n* = 2) and healthy controls (*n* = 2); GEO accession number: PRJNA230906 (see [48] for additional information on the samples). This study analyzed the transcriptomic response of individuals to avian influenza virus (H7N9) infection using whole blood from infected and healthy adults from China using RNA-seq.

The Varicella Zoster infected fibroblast group is composed of six samples of Varicella Zoster Virus (VZV)-infected human dermal fibroblasts cell line (HDF) infected with different strains or vaccines (Suduvax^®^ and Varivix^®^); uninfected cells (*n* = 1), cells infected with wildtype strains (*n* = 2), and cells exposed to the vaccine (*n* = 3); GEO accession number: GSE121385 (see [49] for additional information on the samples). This study focused on analyzing the effects of the wild type VZV and different vaccines using cell cultures; more specifically, primary human dermal fibroblasts infected with wild-type VZV and attenuated varicella strains.

The mononuclear cells group comprises five blood mononuclear cell samples infected with dengue virus (*n* = 4) and one uninfected cell sample (*n* = 1); GEO accession number: GSE98859. This study analyzed changes in the transcriptome of peripheral blood mononuclear cells (PBMCs) when undergoing dengue infection.

The human umbilical vein endothelial cells (HUVECs) group contains samples from enterovirus patients (*n* = 2), coxsackievirus patients (*n* = 2), and healthy controls (*n* = 2); GEO accession number: GSE94551 (see [50] for additional information on the samples).

### 5.2. Data Processing and Statistical Analysis

Quality control of total RNA, libraries preparation and whole transcriptome sequencing (RNA-seq) of the Spanish group (Table S1) were carried out following the protocol described before in [26]. All the RNA-seq raw data files of the present study were preprocessed and normalized as described in [23].

To explore the predictive value of lncRNAs in the context of viral infections, we first detected differentially expressed lncRNAs (meaning transcripts with biotype ‘lncRNA’ according to ENSEMBL) by comparing RV-children (acute and convalescent) versus controls. We focused on the transcripts belonging to the transcript biotype lncRNA according to BiomaRt [51] (Table S2). For this purpose, we employed the R package *Deseq2* using a negative binomial generalized linear model, and including age and sex as covariates of the model [52].

We filtered the lncRNAs to obtain a list of the best candidates according to the following thresholds: *P*-adjusted < 0.05, and log_2_ FoldChange > |2|.

We then used a machine-learning algorithm to select the lncRNAs that are more differentially expressed. We applied the variable selection algorithm *elastic net* implemented in the R package *glmnet* to the list of filtered lncRNA, using the Spanish group (Table S1) as the training group, and the Mexican dataset GSE69529 as the test group. As the elastic net algorithm usually yields a model that includes many genes, (thus rendering its translation to clinical settings difficult) we looked for the most informative genes (minimal signature) among the ones filtered. The parameters needed for the calculation of elastic net were estimated using 10-fold cross-validation. Following a machine learning approach, a single-hidden-layer neural network model was fitted with the R package *nnet* [36], obtaining a 2-transcripts (ENSG00000273149 and ENSG00000254680) viral signature. Viral score (VS) was calculated analogously as in Herberg et al. [13], namely:

VS = log_2_(exprs(ENSG00000273149)) + log_2_(exprs(ENSG00000254680)) (1)

We next evaluated the performance of these markers to detect viral infections in a broader sense. For this purpose, we used other GEO datasets (PRJNA230906, GSE121385, GSE98859, and GSE94551) including controls and viral patient’s RNA-seq data from blood and different cell types samples. While the first study (PRJNA230906,) analyzed the transcriptome of individuals suffering a viral infection, the other three studies focused on analyzing host response to viral infections using cell cultures or single cell types.

To evaluate the diagnostic accuracy of the lncRNAs signature in all the included studies, we used receiver operating characteristic (ROC) curves and the area under the curves (AUC) using the *pROC* [53] package in R. The threshold value, defined as the point on the ROC curve that maximized sensitivity and specificity, was calculated using the R package *OptimalCutPoints* [54]. The calculation of the confidence intervals for sensitivity and specificity was based on a stratified bootstrap resampling.

## Figures and Tables

**Table 1:** Area under the curve (AUC) values and viral score (VS) for different pathogens and studies. Figures in round brackets show the CI95% (calculated using 2000 bootstrap replicates). No expression (NE) means that at least one of the transcripts of the signature was not expressed. CC: Chinese group; SC: Spanish group; VZF: Varicella Zoster infected fibroblasts; MNC: mononuclear cells with dengue, EC: human umbilical vein endothelial cells enterovirus group, MC: Mexican group.

Database		Comparison	*n*	AUC	Sensitivity	Specificity	VS
**2-LncRNA**							
CC	PRJNA230906	virus vs. control	4	1 (1–1)	1	1	2.569
MNC	GSE98859	virus vs. control	5	1 (1–1)	1	1	4.939
EC	GSE94551	virus vs. control	6	1 (1–1)	1	1	4.043
SC	PRJNA325575	virus vs. control	18	1 (1–1)	1	1	7.535
MC	GSE69529	virus vs. control	115	0.891 (0.833–0.950)	0.857	0.838	8.350
MC	GSE69529	virus vs. bacteria	220	0.731 (0.662–0.800)	0.814	0.600	8.113
MC	GSE69529	bacteria vs. control	175	0.707 (0.621–0.793)	0.650	0.743	8.392
VZF	GSE121385	virus vs. control	6	1 (1–1)	1	1	15.015
**2-LncRNA + IFI44L**							
CC	PRJNA230906	virus vs. control	4	1 (1–1)	1	1	1.980
MNC	GSE98859	virus vs. control	5	1 (1–1)	1	1	2.596
EC	GSE94551	virus vs. control	NE	NE	NE	NE	NE
SC	PRJNA325575	virus vs. control	18	1 (1–1)	1	1	3.227
MC	GSE69529	virus vs. control	115	0.857 (0.784–0.929)	0.800	0.850	6.133
MC	GSE69529	virus vs. bacteria	220	0.724 (0.651–0.797)	0.855	0.525	4.101
MC	GSE69529	bacteria vs. control	175	0.723 (0.623–0.824)	0.790	0.714	6.883
VZF	GSE121385	virus vs. control	6	1 (1–1)	1	1	9.670
**2-LncRNA + IFI44L + FAM89A**							
CC	PRJNA230906	virus vs. control	4	1 (1–1)	1	1	2.328
MNC	GSE98859	virus vs. control	5	1 (1–1)	1	1	4.899
EC	GSE94551	virus vs. control	NE	NE	NE	NE	NE
SC	PRJNA325575	virus vs. control	18	1 (1–1)	1	1	11.640
MC	GSE69529	virus vs. control	115	0.850 (0.774–0.927)	0.800	0.863	8.247
MC	GSE69529	virus vs. bacteria	220	0.753 (0.684–0.822)	0.630	0.788	7.469
MC	GSE69529	bacteria vs. control	175	0.681 (0.579–0.783)	0.710	0.714	8.773
VZF	GSE121385	virus vs. control	6	1 (1–1)	1	1	9.679
**FAM89A + IFI44L**							
CC	PRJNA230906	virus vs. control	4	1 (1–1)	1	1	−2.650
MNC	GSE98859	virus vs. control	5	0.625 (0.500–0.875)	1	0.250	−0.091
EC	GSE94551	virus vs. control	NE	NE	NE	NE	NE
SC	PRJNA325575	virus vs. control	18	1 (1–1)	1	1	4.715
MC	GSE69529	virus vs. control	115	0.829 (0.749–0.910)	0.771	0.825	−3.764
MC	GSE69529	virus vs. bacteria	220	0.745 (0.674–0.816)	0.813	0.638	−4.834
MC	GSE69529	bacteria vs. control	175	0.647 (0.544–0.749)	0.748	0.629	−2.924
VZF	GSE121385	virus vs. control	6	1 (1–1)	1	1	−2.270

## Supplementary Materials

The Supplementary Materials are available online at https://www.mdpi.com/1422-0067/21/8/2748/s1.
